# Effects and mechanisms of exosomes in microenvironment angiogenesis in breast cancer: An updated review

**DOI:** 10.32604/or.2024.059113

**Published:** 2025-05-29

**Authors:** JUNPING LIU, FEIRAN GAO, DANTING WANG, RUIXUE ZHOU, CHUNYAN HUANG

**Affiliations:** 1 Department of Blood Transfusion, West China Hospital, Sichuan University, Chengdu, 610041, China; 2 Department of Blood Transfusion, West China Tianfu Hospital, Sichuan University, Chengdu, 610000, China

**Keywords:** Exosomes (EXOs), Breast cancer, miRNAs, Angiogenesis, Notch, Hypoxia

## Abstract

Exosomes (EXOs) play an important role in the progression of breast cancer. EXOs, with a diameter of approximately 100 nm, have a simple structure but diverse functions, and can affect the development of breast cancer through signal transduction and molecular transfer, etc. Angiogenesis provides nutrients for the growth and metastasis of breast cancer and is a crucial part of tumor progression. The mechanism of tumor angiogenesis is complex. The VEGF/VEGFR pathway promotes angiogenesis by regulating the activities of ECs. Hypoxia, a common feature in the tumor microenvironment, as a key regulator, can affect angiogenesis in multiple aspects such as the transfer of miRNAs in EXOs, protein transport, extracellular matrix regulation, and metabolic adaptation. The Notch pathway has a bidirectional regulatory role in breast cancer angiogenesis, and different molecules can promote or inhibit angiogenesis. EXOs secreted by breast cancer cells are rich in angiogenic factors. Components such as proteins and nucleic acids in EXOs can affect the functions and behaviors of vascular ECs, thereby influencing breast cancer angiogenesis. Research on the mechanisms of EXOs in breast cancer angiogenesis is of great significance for tumor treatment. EXOs are expected to become biomarkers for breast cancer diagnosis/prognosis. This research provides potential targets for in-depth understanding of the biological characteristics of breast cancer and the development of new treatment strategies.

## Introduction

The development of breast cancer involves a series of critical biological steps, among which the interaction between cancer cells and surrounding stromal cells is considered a significant issue. Notably, angiogenesis is a key tumorigenic phenomenon in breast cancer progression. Tumor-derived EVs play a role in modulating the favorable microenvironment for cancer cells through their intercellular communication functions. Proteins such as CD63, CD9, heat shock proteins (HSP90 and HSP70), and the Rab family found in EXOs are recognized as specific markers. Factors such as hypoxic microenvironments, survival pressures, and treatments like radiation and chemotherapy can induce tumor cells to secrete EXOs, explaining why tumor cells often secrete more EXOs than normal cells [1]. Vesicle-associated cells, such as ECs, platelet-activated cancer cells, and tumor-derived EXOs, can promote coagulation leading to an inflammatory state, shaping the tumor microenvironment, including extravasation, microthrombi formation, platelet aggregation, and metastasis. EXOs play a major role in breast cancer progression, particularly cancer-derived EXOs that influence ECs. ECs themselves play various roles, such as promoting tumor angiogenesis, causing loss of endothelial vascular barriers upon binding with ECs, followed by endothelial-to-mesenchymal transition and extracellular matrix remodeling. Therefore, intercellular communication via EXOs between breast tumor cells and ECs may be a critical target for controlling breast cancer progression [2]. EXOs derived from breast tumor cells not only directly promote angiogenesis by activating the VEGF/VEGFR pathway [3], but also indirectly through pathways such as hypoxia, Notch [4], MAPK/ERK [5], and AMPK [6]. The proteins, lncRNAs, miRNAs, and other contents within these EXOs are crucial during the process of tumor initiation and development.

In this article, the role of EXOs in angiogenesis within the breast cancer microenvironment and its molecular mechanisms are thoroughly explored. Specifically, the study aims to disclose the crucial role of EXOs in breast cancer angiogenesis. By systematically analyzing the secretion of EXOs in different breast cancer cell lines and their impact on angiogenesis, the core role of EXOs in the progression of breast cancer will be determined. To elucidate the EXO-mediated signal pathways, this study will investigate in detail how EXOs regulate breast cancer angiogenesis through signal pathways such as VEGF/VEGFR, Notch, and AMPK, and reveal the specific mechanisms of these pathways in EXO-mediated angiogenesis. To further evaluate the potential of EXOs as biomarkers for breast cancer, based on an in-depth understanding of the mechanism of action of EXOs in breast cancer angiogenesis, this study will propose new treatment strategies and potential drug targets, providing a theoretical basis for the precision treatment of breast cancer. In summary, this study aims to comprehensively reveal the complex roles and mechanisms of EXOs in breast cancer angiogenesis, providing new perspectives and methods for the diagnosis, treatment, and prognosis of breast cancer.

## An Overview of EXOs

Extracellular vesicles have been discovered for over 30 years, existing in various forms. Based on their diameter, they are categorized into EXOs, microvesicles, apoptotic bodies, and tumor-derived vesicles, among others [7]. Unlike other vesicles that are released through the plasma membrane, EXOs are released via an endocytic pathway involving numerous complex pathways for their formation and secretion. The process of EXO release into the extracellular space involves: the formation of early endosomes, budding of these early endosomes to form multivesicular bodies, and the final fusion of multivesicular bodies with the plasma membrane, releasing their contents as EXOs [8]. Escola et al. [9] discovered that EXOs are highly enriched in certain tetraspanins (CD81, CD82, CD37, CD63). Among these proteins, CD81 is the most abundant, primarily localized to the plasma membrane, while CD63, which is rich in endosomes, is relatively less abundant in EXOs. Since the first reports, CD81 and CD63 have been the most commonly used markers for EXOs, along with other tetraspanins, especially CD9 [10]. EXOs carry various peripheral surface proteins and extracellular matrix proteins, many of which are involved in signal transduction. These include several Wnt proteins [11], TNF [12], and a range of other surface signaling molecules, further indicating that EXOs can act as carriers of autocrine and paracrine signals. In addition to proteins, EXOs are rich in nucleic acids, facilitating the transfer of genetic material between cells, tissues, and organs. Investigations into exosomal RNA have revealed an abundance of small non-coding RNAs, including siRNA [13], microRNA [14], tRNA [15], vault RNAs, and fragment RNAs [16]. Overall, EXOs often contain a specific set of RNAs, which are related to the RNA profile of their cells of origin.

## Mechanisms of Tumor Angiogenesis

The formation of new blood vessels, especially in hypoxic environments, is crucial for tumor cells to continue proliferating. Angiogenesis is the process where ECs bud and elongate to form small blood vessels. It is essential for solid tumor growth and distant metastasis. The structure of tumor neovasculature is unique, lacking a complete vessel wall and smooth muscle, composed only of ECs and a basement membrane. These newly formed capillaries supply oxygen and nutrients to tumors and remove metabolic waste. Moreover, increased vascular permeability and a fragile basement membrane facilitate tumor cell invasion and metastasis. Tumor angiogenesis is a critical step in tumor growth and metastasis, involving pathways such as VEGF/VEGFR, hypoxia, and Notch signaling. The VEGF/VEGFR pathway promotes tumor angiogenesis by regulating ECs activity and vascular stability, thereby supporting tumor growth and metastasis by providing nutrients and oxygen. Hypoxia, a common feature of the tumor microenvironment, stimulates tumor cells to produce VEGF, further promoting tumor angiogenesis. Hypoxia also activates other pathways like Notch signaling, which further enhances tumor angiogenesis by inducing differentiation of ECs to form new vessels, supplying nutrients and oxygen to the tumor. In summary, angiogenesis plays a significant role in the malignant progression of tumors. Further understanding of these mechanisms is crucial for clinical oncology treatments.

### VEGF/VEGFR pathway in angiogenesis

Well-known pro-angiogenic factors include vascular endothelial growth factor (VEGF), bFGF, EGF, PDGF, insulin-like growth factor, TGF, and angiopoietin [17]. Among these, VEGF plays a key role in promoting ECs mitosis and migration, ultimately leading to angiogenesis. VEGF is a homodimeric glycoprotein family that includes VEGF-A, VEGF-B, VEGF-C, VEGF-D, and PLGF in mammals [18]. Specifically, VEGF-A was the first identified and characterized angiogenic factor in the VEGF family. VEGF-A exists in various isoforms such as VEGF-A121, VEGF-A165, VEGF-A189, and VEGF-A206 [19]. Among these, VEGF-A165 is the most active in angiogenesis, crucial not only in physiological processes like embryonic vascular development and skeletal morphogenesis but also in pathological angiogenesis, including tumor cell metastasis [20,21]. Members of the VEGF family bind to three tyrosine kinase receptors—VEGFR1, VEGFR2, and VEGFR3—as well as two co-receptors NRP1 and NRP2. There exists a complex pattern of cross-binding between VEGF and its receptors, activating different receptors to induce angiogenesis. Specifically, VEGF-A binds to both VEGFR1 and VEGFR2, VEGF-B and PLGF bind only to VEGFR1, while VEGF-C and VEGF-D bind to both VEGFR2 and VEGFR3 [22–24]. VEGFR1 and VEGFR2 are predominantly expressed on vascular ECs but are also expressed in certain cancer cells. VEGFR2, as the primary receptor in VEGF signaling, has been extensively studied. Binding of VEGF to VEGFR2 triggers robust downstream signaling, promoting EC proliferation, migration, and tubule formation [25]. However, the exact mechanisms of VEGFR1 signaling remain incompletely understood to date. The regulation of angiogenesis is particularly intriguing in oncology, as new vessels supply nutrients to tumor cells, promoting their growth [26]. In clinical practice, drugs with various mechanisms are commonly used to inhibit VEGF/VEGFR and thereby suppress tumor progression [27]. This includes monoclonal antibodies like bevacizumab, which specifically targets VEGF-A, blocking the angiogenic pathway in ECs [28]. Additionally, multi-targeted small molecule inhibitors such as sorafenib, sunitinib, pazopanib, and vandetanib can inhibit multiple tyrosine kinases including VEGFR, thereby halting angiogenesis. Inhibitors of the VEGF pathway are employed in various cancers including renal cell carcinoma [29], colorectal cancer [30], ovarian cancer [31], and non-small cell lung cancer [27].

### Hypoxia affects angiogenesis through EXOs

**Figure 1:**
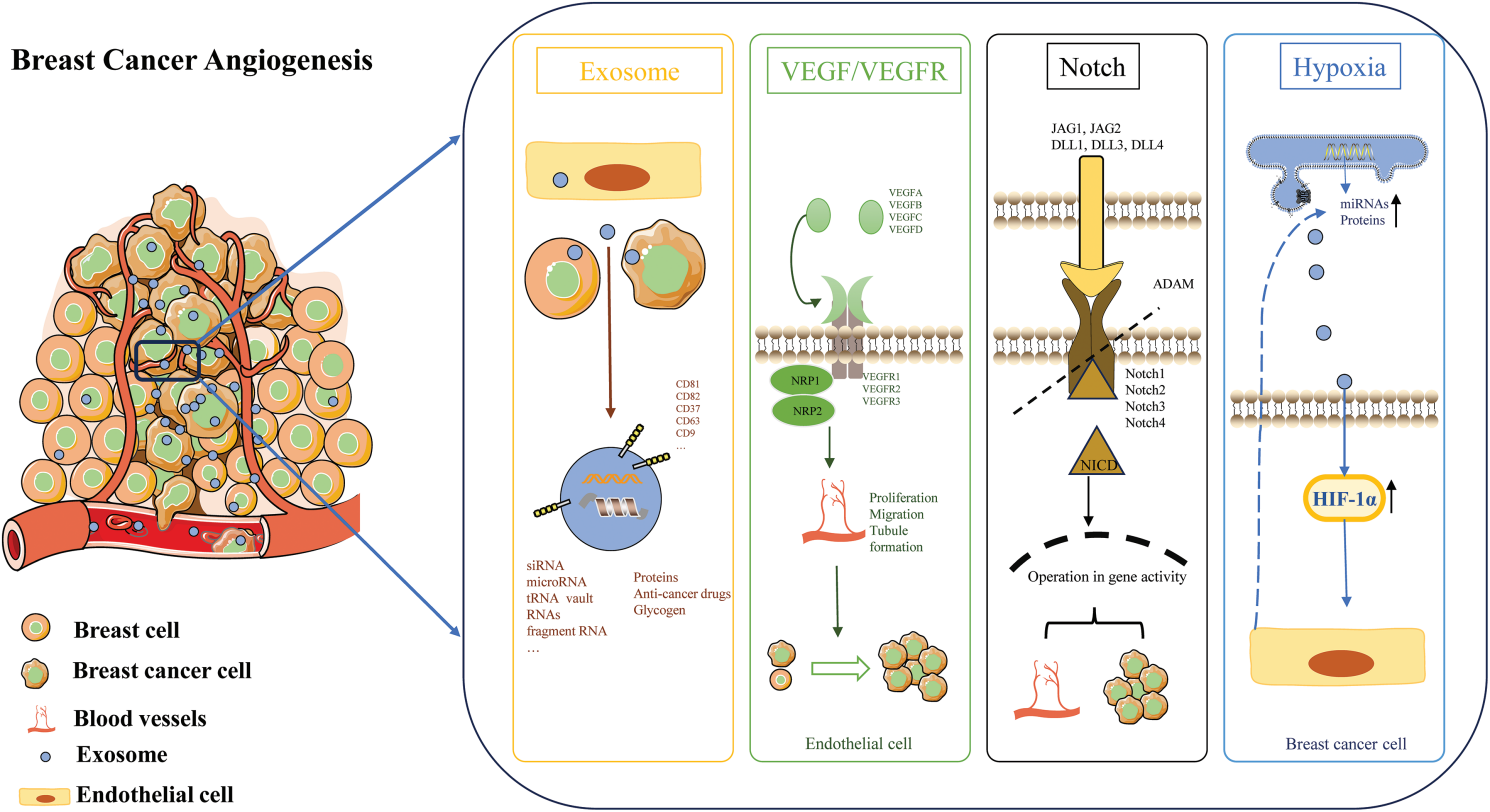
Angiogenesis in the breast cancer microenvironment.

**Figure 2:**
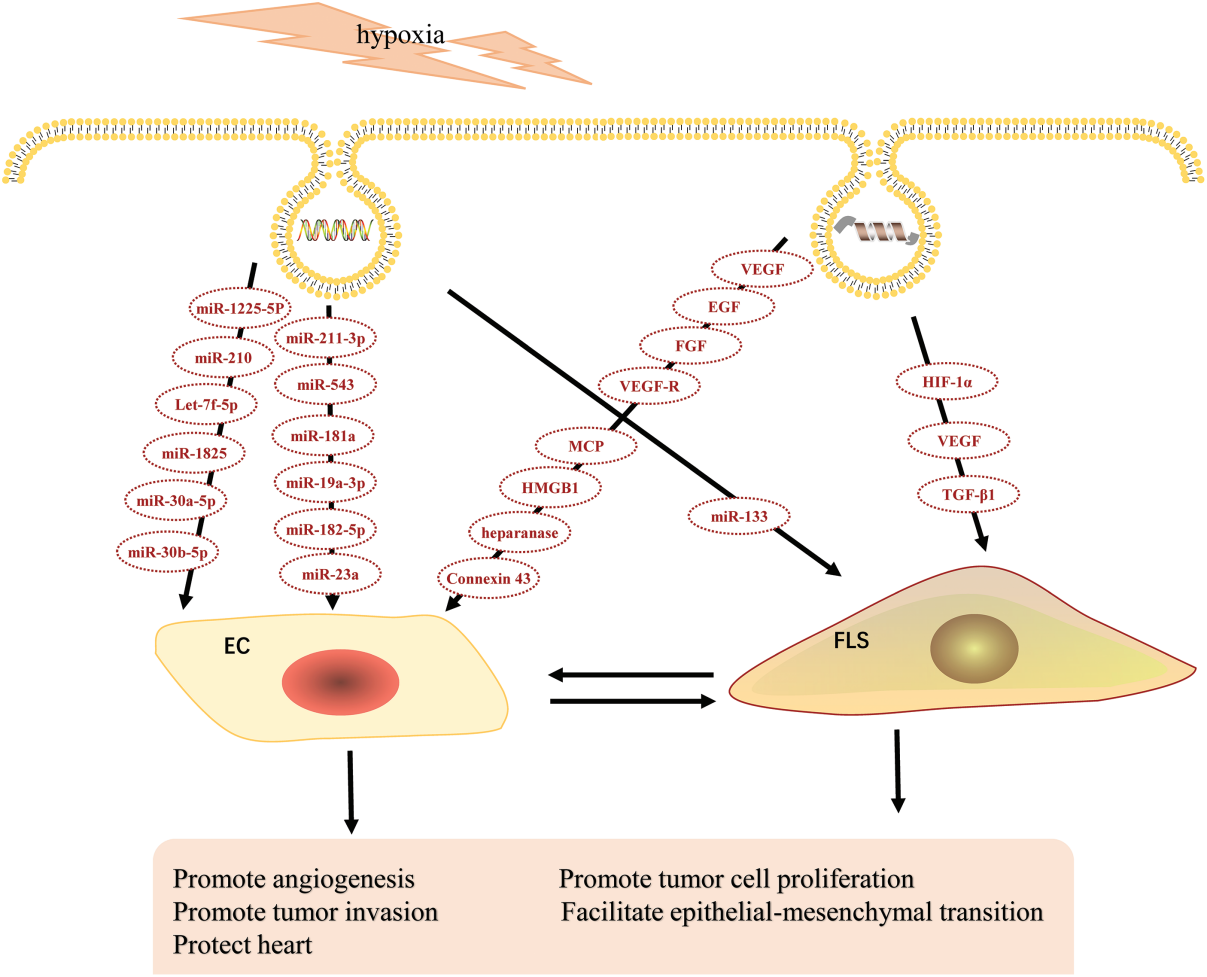
Effects of hypoxic-derived EXOs on angiogenesis.

Hypoxia is a condition where tissues or cells experience insufficient oxygen supply, triggering a series of physiological and pathological processes. In angiogenesis, hypoxia serves as a critical regulatory factor. Recent studies have shown that EXOs have a significant impact on angiogenesis under hypoxic conditions (Fig. 1). Under hypoxia, the composition and quantity of EXOs secreted by cells undergo significant changes, influencing the process of angiogenesis. The main mechanisms through which hypoxia affects angiogenesis via EXOs include regulating the transfer of miRNAs. Under hypoxic conditions, EXOs secreted by cells contain specific miRNAs such as miR-1225-5p, which is enriched in hypoxic EXOs and indirectly affects the malignant phenotype of ovarian cancer [32]. Similarly, miR-210, present in EXOs from hypoxia-preconditioned cells, promotes angiogenesis in dental pulp tissue by transferring let-7f-5p and miR-210-3p [33]. miR-1825 [34], miR-30a-5p [35], miR-30b-5p [36], miR-211-3p [37], miR-543 [38], miR-181a [39], miR-19a-3p [40], miR-182-5p [41], miR-23a [42], whose increased secretion in hypoxia alters the angiogenic potential of ECs, these miRNAs can be internalized by ECs to regulate the expression of angiogenesis-related genes. Hypoxic EXOs carry microRNAs that not only affect angiogenesis in tumor diseases but also promote angiogenesis in myocardial infarction, including miR-211-3p, miR-543, and miR-19a-3p. Hypoxia also affects angiogenesis through changes in the transport of proteins via EXOs. Under hypoxic conditions, EXOs may contain pro-angiogenic proteins such as VEGF, EGF, FGF and their receptors (VEGF-R2, VEGF-R3), and MCP-2, MCP-4, which are absorbed by ECs to directly promote their proliferation, migration, and lumen formation [43]. The expression of HMGB1 in EXOs induced by hypoxia increases, activating the JNK signaling pathway and inducing the expression of hypoxia-inducible factor-1α/vascular endothelial growth factor, thereby enhancing angiogenesis in HUVECs [44]. In addition, EXOs from hypoxic tumor cells, carrying elevated levels of heparanase cargo, significantly promote the formation of ECs tubes, consistent with the known role of heparanase in promoting angiogenesis [45]. Similar to this, connexin 43 is loaded more in hypoxic EXOs, which can directly induce proliferation and tube formation of vascular ECs [46]. Hypoxia regulates angiogenesis through EXOs by modulating the ECM [47]. Under hypoxic conditions, EXOs transport enzymes such as MMPs, which can alter the composition and structure of the ECM to provide a more suitable environment for the growth of new blood vessels [48]. Research has found that hypoxic EXOs carry more miR-133, leading to excessive activation and proliferation of fibroblasts and the secretion of excessive extracellular matrix components, resulting in normal cardiac structural damage and heart fibrosis [49]. Moreover, under hypoxic conditions, EXOs can alter the metabolic status of target cells by transferring metabolic regulators, enabling them to adapt more effectively to low oxygen environments [50]. For instance, HIF-1α in hypoxic EXOs regulates glycolysis-related enzymes, promotes cellular metabolic reprogramming, increases ATP generation, and supports ECs proliferation and migration. In conclusion, hypoxia exerts multifaceted effects on angiogenesis through EXOs, including regulation of gene expression, protein transport, ECM modulation, and metabolic adaptation. These mechanisms collectively promote angiogenesis, supporting tissue survival and functional recovery under hypoxic conditions. Future insights into the specific mechanisms of exosomal regulation under hypoxia hold promise for novel strategies and targets in breast cancer therapy (Fig. 2).

Exosomes play a crucial role in breast cancer angiogenesis, a process vital for tumor growth and metastasis. Breast cancer-derived exosomes are small extracellular vesicles that carry a variety of bioactive molecules, such as proteins, miRNAs, and glycogen. The VEGF signaling pathway plays a crucial role in angiogenesis and vascular development. VEGF is a family of growth factors, including VEGFA, VEGFB, VEGFC, VEGFD. VEGF binds to specific tyrosine kinase receptors on the surface of endothelial cells. The main receptors are VEGFR1, VEGFR2, and VEGFR3. Hypoxia, a common condition in solid tumors including breast cancer, is a key factor influencing exosome secretion. Under hypoxic conditions, breast cancer cells are stimulated to release more exosomes. These hypoxia-induced exosomes can then participate in the regulation of angiogenesis. In breast cancer angiogenesis, the Notch signaling pathway can be activated by exosome-mediated mechanisms. Activation of the Notch pathway can lead to endothelial cell proliferation, migration, and tube formation, which are essential steps in angiogenesis. By modulating the Notch signaling pathway under hypoxic conditions, exosomes promote breast cancer angiogenesis, ultimately facilitating tumor progression and metastasis.

### Notch pathway in breast cancer angiogenesis

The Notch signaling pathway is a highly conserved intercellular communication mechanism that plays a crucial role in the development of various organisms. Activation of the Notch pathway occurs through interactions between Notch receptors on the surface of adjacent cells and their ligands. In mammals, the Notch receptor family includes four members, Notch1 through Notch4, while the ligand family comprises two subfamilies, Delta-like (DLL1, DLL3, DLL4) and Jagged (JAG1, JAG2). The ligand Dll-4, for instance, regulates vascular sprouting and branching by influencing the number of tip cells. Recent studies have increasingly focused on the role of the Notch pathway in angiogenesis in breast cancer. As shown in Table 1, with the exception of JAG2, various Notch molecules participate in either promoting or inhibiting angiogenesis processes in breast cancer. Specifically, Notch1/Notch3/Notch4/DLL1/DLL3/JAG1 exhibit promoting effects, while Notch2 acts as an inhibitor of angiogenesis. Interestingly, DLL4 appears to have context-dependent roles in angiogenesis. Research by Sheldon et al. [51] found that in U87-GM glioblastoma cells overexpressing Dll-4, Dll-4 is packaged into EXOs and transported to ECs, where it inhibits Notch signaling, leading to increased vascular branching and density. Similarly, elevated Dll4 signals in vessels of cancers like breast and bladder suggest a role in suppressing excessive vascular proliferation, reducing vessel numbers, and promoting luminal expansion to enhance tumor cell blood supply. From a therapeutic perspective, blocking DLL4 signaling is akin to cutting off the tumor blood supply, thereby inhibiting tumor growth. The DLL4-Notch signaling pathway typically operates downstream of VEGF and is crucial in angiogenesis in breast cancer. Inhibiting the DLL4-Notch pathway can promote excessive proliferation of ECs, resulting in the formation of non-functional neovessels [52]. Moreover, inhibitors of DLL4 like HB-32 and H3L2 have shown significant efficacy in inhibiting solid tumors in breast cancer research [53] Additionally, studies have linked JAG1 expression to vessel numbers in tumors, and interactions between JAG1/Notch3 and VEGF may play a role in angiogenesis in triple-negative breast cancer [54,55]. However, further experimental research is needed to clarify how JAG1 contributes to the progression of triple-negative breast cancer.

**Table 1:** Dysregulation of Notch signaling in angiogenesis in breast cancer

Notch receptors or ligands	Types of breast cancer	Molecules of crosstalk	Effect on angiogenesis
Notch1	MCF-7, MDA-MB-231	EXOs/miR-423-5p [56], phosphoserine aminotransferase 1 (PSAT1)/β-catenin/NICD1 [57], non-receptor tyrosine kinase PYK2 [58]	Promote
Notch2	ZR-75-1, MDA-MB-231, and T47D	MINAR1 [59], Withaferin A [60]	Inhibit
Notch3	TNBC	Jagged1/VEGF [61]	Unclear
Notch4	TNBC	Newly developed anti-Notch4 antibody: E7011 [62]	Promote
DLL1	MCF7 and T47D	Estrogen receptor (Erα)/HES1 and HEY1 [63,64]	Promote
DLL3	MCF7	Notch4/VEGFR3 [65]	Promote
DLL4	MCF7 and MDA-MB-231	linc-OIP5/YAP1/JAG1 [55], HB-32/VEGF [53], H3L2 [52]	Promote/Inhibit
JAG1	TNBC, MDA-MB-231	Notch3/VEGF, lysine demethylase 2A (KDM2A) Notch1/HEY1 [66],	Promote
JAG2	No study	No study	No study

### EXOs, microRNAs, VEGF and NOTCH: interactions in breast cancer angiogenesis

As an important carrier for intercellular communication, EXOs play a crucial role in the context of breast cancer angiogenesis through their internal components. This role goes beyond simple substance transportation and extends to the transmission of signals related to breast cancer angiogenesis. MicroRNAs show a definite regulatory ability on VEGF in breast cancer angiogenesis. They can specifically bind to the 3′-UTR region of VEGF mRNA, which is a highly precise regulatory site. By inhibiting the translation of VEGF mRNA or inducing its degradation, the synthesis of VEGF protein is subsequently reduced. For example, miR-573 can decrease the expression of VEGF, thereby inhibiting angiogenesis [67]. This regulation of VEGF protein synthesis further affects angiogenesis-related processes such as the proliferation, migration of vascular ECs and blood vessel formation, highlighting that the regulation of VEGF by miRNAs is a key link affecting breast cancer angiogenesis. That is, miRNAs indirectly affect the entire angiogenesis process by regulating this key pro-angiogenesis factor VEGF. In terms of the Notch signaling pathway, microRNAs can regulate key molecules in it, such as Notch receptors and ligands. In the process of breast cancer angiogenesis, the Notch signaling pathway has complex functions (different Notch molecules can play promoting or inhibiting roles in angiogenesis). MiRNAs affect the activity of the entire pathway by regulating the expression of these molecules. In other words, the regulation of the Notch signaling pathway by microRNAs is not a simple linear relationship but is affected by multiple factors, forming a complex network in the process of breast cancer angiogenesis.

Different miRNAs may have different regulatory effects on different molecules in the Notch signaling pathway, thus causing different impacts on angiogenesis. Some miRNAs can target various components of the Notch pathway at different levels. For instance, miR-338-5p was found to be able to affect the proliferation and metastasis of breast cancer by acting on the transcription factor ETS1 of Notch1, and this has been demonstrated in breast cancer tissues as well as xenograft tumor models [68]. Besides affecting the transcription factors of Notch, certain miRNAs have been found to directly bind to the mRNA of Notch receptors or their ligands, thereby regulating their expression levels. Combination therapy of miRNA-34a with doxorubicin was found to synergistically inhibit the progression of doxorubicin-resistant breast cancer by inhibiting Notch/NF-κB signaling [69]. In addition, the forced expression of the tumor-suppressive miR-181c-5p negatively regulates the oncogenic Notch1 signaling in TNBC [70]. In the development of estrogen-dependent cancers (such as breast cancer, endometrial cancer, and ovarian cancer), miR-765 is found to be significantly down-regulated, and it indirectly affects the Notch signaling pathway through EXOs released by CD8+ T cells, showing great potential in tumor immunotherapy [71]. Does Notch affect the biological activity of miRNA? Unfortunately, no relevant research has been found in breast cancer so far. However, some scholars have found in embryonic development research that perturbations in the Delta/Notch signaling pathway do not cause significant changes in miRNA levels. In breast cancer cells, the dysregulation of this miRNA-Notch pathway relationship can impact cell proliferation, differentiation, and angiogenesis. This disruption can then contribute to the development and progression of breast cancer by promoting abnormal cell growth and survival.

Overall, in the process of breast cancer angiogenesis, miRNAs contained in EXOs play an indispensable role by regulating the VEGF and Notch signaling pathways. The interactions among EXOs, microRNAs, VEGF and Notch form a complex regulatory network. The complexity and diversity of these interactions need to be further explored, which is helpful for in-depth understanding of the molecular mechanisms of breast cancer angiogenesis and may open up new targets and strategies for breast cancer treatment.

## EXOs in Breast Cancer Angiogenesis

EXOs secreted by breast cancer cells are rich in angiogenic factors, thus inducing angiogenesis in various cancers and tumor microenvironments. EXOs can affect the function and behavior of vascular ECs in several ways. First, they can stimulate ECs proliferation and migration, laying the foundation for angiogenesis. Second, they can regulate ECs permeability, facilitating the exchange of substances necessary for angiogenesis. Additionally, EXOs can alter the tumor microenvironment to make it more conducive to angiogenesis. They can induce changes in immune cells, suppress immune surveillance, and promote tumor growth and angiogenesis. When EXOs from TNBC are co-cultured with HUVECs, they promote the proliferation of HUVECs in a time- and dose-dependent manner [72]. In summary, EXOs play multiple roles in breast cancer angiogenesis and are important factors in breast cancer development and metastasis. In-depth studies on the relationship between EXOs and angiogenesis will provide new targets and strategies for breast cancer treatment. A question worth exploring is which components within EXOs influence angiogenesis.

### Proteins in EXOs affecting angiogenesis in breast cancer

EXOs can directly deliver VEGF, TGF-β, CXCR4, and other angiogenesis-related growth factors to promote angiogenesis [73–75]. EXOs play a key role in the transport of Cav-1 between the microenvironments of primary breast cancer and metastatic organs [76]. Cav-1 in breast cancer-derived EXOs can act as a signaling molecule, regulating the formation of PMN in lung epithelial cells and inhibiting the PTEN/CCL2/VEGF-A signaling pathway in lung macrophages, thus promoting their M2 polarization and angiogenesis. Similarly, EXO-transported Wnt7a increases the formation of breast cancer cell spheroids by activating the PI3K/Akt/mTOR signaling pathway, promoting fibroblast infiltration and angiogenesis, while the absence of Wnt7a significantly reduces angiogenesis [77]. EXO-mediated transfer of oncogenic EGFR can effectively disrupt tight junctions and the integrity of ECs barriers, promoting tumor angiogenic sprouting [78]. Unlike the internal transportation method of EXOs, recent studies have found that EPHA2 can be expressed on the surface of EXOs [6]. EPHA2, a member of the EPH receptor tyrosine kinase family, is normally expressed on the membrane of epithelial cells and interacts with its ligand Ephrin A1. EXOs from breast cancer cells are rich in EPHA2 and can transfer it to ECs. EPHA2 in EXOs can promote angiogenesis through AMPK signaling, enhancing the ability to form new blood vessels. The promotion of breast cancer angiogenesis by EXOs is often closely related to the formation of pre-metastatic niches, including the transformation of fibroblasts and the infiltration of inflammatory factors.

Besides proteins, lipids carried by EXOs also play a role in promoting angiogenesis. DGs accumulated in breast cancer EXOs can induce phosphorylation of PKD/PKC and related PKC pathways in HUVECs. DGs work in coordination with other components of EVs to stimulate angiogenesis [79].

### Nucleic acids in EXOs affecting angiogenesis in breast cancer

EXO-carried miRNAs generally do not directly affect the tubulogenesis ability of ECs but rather influence angiogenesis-related proteins such as VEGF and EGFR. For example, miR-573 can reduce the expression of VEGF and CD146, thus decreasing angiogenesis and exerting its anti-breast cancer effects [67]. EXOs derived from adipose mesenchymal stem cells deliver miR-218 to breast cancer cells, where miR-218 can inhibit angiogenesis and EMT by targeting Runx2 and Rictor, preventing breast cancer progression [80]. Other miRNAs like miR-329 [3], miR-124-3p [81], and miR-145 [82] also target breast cancer angiogenesis. Studies have found that EXOs from trastuzumab-resistant cells have significantly higher levels of miR-146a-5p. Further experiments show that co-culture with resistant cell EXOs reduces sensitivity and enhances the migration and angiogenesis abilities of sensitive cells [83]. While miR-573 and miR-218 inhibit breast cancer angiogenesis, miR-146a-5p promotes it through EXOs.

In addition to miRNAs, lncRNA SNHG1 acts as a tumor promoter in various cancers, including colorectal cancer, breast cancer, and prostate cancer. Exosomal SNHG1 from hypoxic breast cancer cells can promote tumor angiogenesis and growth by regulating the miR-216b-5p/JAK2 axis [84]. A novel lncRNA, AC073352.1, may be packaged into EXOs through binding with YBX1 in breast cancer cells, leading to angiogenesis [85].

In conclusion, EXOs promote angiogenesis by transmitting angiogenic proteins, lncRNAs, and miRNAs for intercellular signaling (Table 2). Under normoxic conditions, miRNAs in tumor EXOs mainly regulate angiogenesis by targeting VEGF, while under hypoxic conditions, they influence angiogenesis through upstream factors of VEGF. Considering the importance of hypoxia and angiogenesis in tumor progression, inhibiting the effects of tumor EXOs on VEGF might become a key mechanism for counteracting malignant tumor progression. The content of EXOs is far more extensive than mentioned in this article. Many more biomolecules within EXOs function as breast cancer biomarkers yet to be discovered. Future research may uncover additional breast cancer biomarkers, leading to critical breakthroughs in tumor treatment.

**Table 2:** The role of EXOs in angiogenesis in breast cancer

EXO content	Effects in angiogenesis
Proteins	VEGF, TGF-β, CXCR4 [73–75], Cav-1 [76], Wnt7a [77], EGFR [78] and EPHA2 [6] enhance vascular formation and influence tumor microenvironment dynamics.
Lipids	Exosomal lipids like DGs [79] in breast cancer EXOs induce PKD/PKC phosphorylation in HUVECs, enhancing angiogenesis.
Nucleic acids	miR-573 [67], miR-218 [80], miR-329 [3], miR-124-3p [81], miR-145 [82], miR-146a-5p [83], LncRNA SNHG1 [84] and lncRNA AC073352.1 [85] regulate angiogenesis-related gene expression by targeting VEGF, EGFR, and other proteins.

### EXOs in breast cancer treatment: potential and challenges

EXOs have good biocompatibility and low immunogenicity and can avoid the attack of the immune system. They can cross biological membranes and deliver the drugs or therapeutic molecules they carry into tumor cells. For example, EXOs can enter tumor cells through endocytosis and then release the carried drugs inside the cells. miRNA antagomir was loaded into EXOs extracted from conditioned medium by electroporation. Then, MDA-MB-231 cells were treated with EXOs loaded with antagomir, and it was found that EXOs can be effectively used as carriers for oligonucleotides such as miRNA mimics and antagomir in targeted therapy [86]. Through engineering EXOs, their targeting ability and drug—loading capacity can be enhanced. For example, tumor—specific ligands (such as folic acid, RGD peptides, etc.) can be modified on the surface of EXOs, enabling them to specifically recognize and bind to breast cancer cells [87]. Meanwhile, chemotherapeutic drugs, gene therapy vectors (such as siRNA, shRNA, etc.) or immunomodulatory molecules can be loaded into EXOs to achieve precise treatment of breast cancer. Some studies have developed biomimetic nanoparticles to improve the delivery of drugs to the breast cancer metastatic microenvironment [88]. Nanoparticles can protect siRNA from degradation and exhibit excellent biocompatibility, showing a significant gene-silencing effect that significantly inhibits the growth of malignant breast cancer cells. Therapeutic strategies developed based on potential targets in EXO research are expected to improve the survival rate and quality of life of breast cancer patients. For example, personalized medicine can select the most suitable targeted therapeutic drugs according to the characteristics of EXOs in patients’ tumor tissues. In addition, EXOs as drug-delivery carriers can improve the efficacy of existing drugs and reduce side effects. However, EXO research also faces some challenges in clinical applications. For example, the methods for EXO isolation, purification, and identification need to be further standardized to ensure their quality and consistency. In addition, the *in vivo* distribution and metabolic mechanisms of EXOs are not fully understood, and more research is needed to optimize their application in clinical treatment.

Cancer-derived EVs in body fluids hold promise as biomarkers for cancer diagnosis. Now, a great number of studies have proven that EXOs in body fluids can be extracted and their contents and surface proteins can be detected. An isolation-analysis integrated cancer diagnosis platform based on DEP-ELISA technology can be applied to EVs-based liquid biopsies. The diagnostic accuracies for breast cancer is 94.2% [89]. Continuous monitoring of surface protein markers on EXOs in the blood could offer valuable insights for breast cancer diagnosis. Wang, et al. developed a method of integrated centrifugal disk chip (CD chip) EXO enrichment directly from whole blood followed by a colorimetric visualization strategy for multiplex analysis [90]. A new EXO carbohydrate marker, TF-Ag-α (Galβ1-3GalNAc-α), has an accuracy rate of over 95% in detecting lung cancer and breast cancer and can be used for cancer screening and early detection [91]. Serum exosomal miR-200c could differentiate between patients with and without breast cancer disease and could be used as an early diagnostic marker for breast cancer disease [92]. Given that the up-regulation of miR-106b is closely associated with several malignant tumors, sensitive and accurate detection of miR-106b in EXOs can serve as an important biomarker for breast cancer diagnosis [93]. All in all, EXOs can evade immune system attacks and deliver therapeutic molecules into tumor cells. They can be engineered to enhance targeting and drug-loading capacities, such as by modifying their surfaces with tumor-specific ligands. EXOs have been successfully used as carriers for oligonucleotides like miRNA mimics and antagomir in targeted therapy. EXO research holds potential for improving survival rates and quality of life for breast cancer patients through personalized medicine and enhanced drug efficacy. EXOs in body fluids also show promise as biomarkers for cancer diagnosis, with high accuracy rates in breast cancer, and can be used for early detection and continuous monitoring of disease progression.

## Conclusion

In recent years, research on the VEGF/VEGFR pathway, hypoxia, and Notch signaling pathways in tumor angiogenesis has advanced significantly. Through experimental studies, scientists have elucidated the specific mechanisms of these pathways in tumor angiogenesis, providing new targets for tumor diagnosis and treatment. Inhibitors and antibodies targeting these pathways are continuously being developed, offering new hope for cancer treatment. However, the mechanisms of tumor angiogenesis remain complex and diverse, requiring further research to reveal more details and provide more effective strategies for tumor prevention and treatment.

Angiogenesis is a complex biological process involving multiple regulatory factors, with the VEGF playing a crucial role. Upon binding with its receptor VEGFR2, VEGF activates a series of signaling pathways to promote ECs proliferation, migration, and the formation of vascular lumens. In oncology, inhibition of the VEGF/VEGFR pathway effectively blocks tumor angiogenesis, thereby suppressing tumor growth and dissemination. Treatment strategies include monoclonal antibodies like bevacizumab, which specifically bind to VEGF-A, as well as small molecule multi-target inhibitors such as sorafenib and sunitinib, which exert anti-tumor effects by targeting VEGFR and other tyrosine kinases. Hypoxia is a critical regulator in angiogenesis. Recent studies indicate that extracellular vesicles released by cells under hypoxic conditions play a significant role in angiogenesis. These vesicles contain specific miRNAs (e.g., miR-1225-5p, miR-210) and angiogenic proteins (like VEGF), which can be taken up by target cells, thereby influencing gene expression, protein transport, extracellular matrix, and metabolic status to promote ECs proliferation, migration, and vascular lumen formation. The Notch signaling pathway regulates vascular sprouting and branching by affecting tip cell numbers. In tumors such as breast cancer, various members of the Notch pathway, including Notch1, Notch3, DLL1, and DLL3, have been implicated in either promoting or inhibiting angiogenesis. Particularly, DLL4 expression correlates closely with vascular density in tumors; its inhibition can reduce the formation of functional vessels, thereby suppressing tumor vascular supply and growth. Hence, the Notch signaling pathway holds significant research and therapeutic value in regulating tumor angiogenesis, potentially serving as a novel therapeutic target.

EXOs play multiple crucial roles in promoting breast cancer angiogenesis. By transmitting angiogenesis-related growth factors such as VEGF, TGF-β, and CXCR4, they directly influence blood vessel formation. Additionally, Cav-1 and Wnt7a in EXOs released by breast cancer cells enhance tumor angiogenesis through different signaling pathways. The discovery of EPHA2 on the surface of EXOs reveals its mechanism in promoting angiogenesis in ECs via the AMPK signaling pathway. Moreover, EXOs regulate the expression of angiogenesis-related genes through miRNAs such as miR-573, miR-218, and miR-146a-5p, further impacting breast cancer angiogenesis and metastasis. Overall, EXOs play a significant role in intercellular signaling between breast cancer and ECs by transmitting proteins, lipids, and nucleic acids, driving breast cancer angiogenesis and pathophysiological processes. Given the critical role of EXOs in tumor progression and angiogenesis as an essential factor in breast cancer metastasis, it is meaningful to study the mechanisms of EXOs in breast cancer angiogenesis. More importantly, EXOs are present in most body fluids and can serve as diagnostic/prognostic biomarkers for breast cancer.

Although this article provides a detailed review regarding the roles and mechanisms of exosomes in breast cancer angiogenesis, there are still some limitations. Firstly, current research mainly focuses on cell cultures and animal models and lacks verification from large-scale clinical samples. This restricts the applicability of the research results in actual clinical applications, and more clinical sample studies are required in the future to support the application of exosomes in breast cancer treatment. In addition, Notch molecules not only influence the malignant progression of breast cancer through angiogenesis, but also can affect breast cancer by regulating the apoptosis-signaling pathway, influencing breast cancer stem cell characteristics, and modulating cell-cycle-related proteins, etc. Therefore, whether there are associations between angiogenesis and these aspects still needs further discussion. Finally, the mechanism research on exosomes, angiogenesis, and breast cancer is not yet mature. Due to insufficient research on other non-coding RNAs, such as lncRNAs, piRNA, circRNAs, tRNA, snRNA, etc., this article only provides a relatively detailed review on the roles of miRNA in breast cancer exosomes. These limitations need to be overcome in future research to understand the roles and mechanisms of exosomes in breast cancer angiogenesis more comprehensively and in-depth. Research on EXOs in the tumor angiogenesis microenvironment is still in its early stages, and further elucidation of the exact role of EXOs in breast cancer microenvironment angiogenesis is necessary.

## Data Availability

Data sharing not applicable to this article as no datasets were generated or analyzed during the current study.

## Abbreviations

AKT: Protein kinase B
AMPK: AMP activated Protein Kinase
bFGF: Basic Fibroblast Growth Factor
Cav-1: Caveolin-1
CCL2: Chemokine (C–C motif) Ligand 2
CXCR4: CXC chemokine receptor 4
DEP: Dielectrophoresis
DGs: Diacylglycerols
Dll1: Delta-like Ligand 1
Dll3: Delta-like Ligand 3
Dll4: Delta-like Ligand 4
EC: Endothelial Cell
ECM: Extracellular Matrix
EGF: Epidermal Growth Factor
EMT: Epithelial–Mesenchymal Transition
EPHA2: EPH receptor A2
ERK: Extracellular signal-regulated Kinase
EVs: Extracellular Vesicles
EXO: Exosome
FLS: Fibroblast-like synoviocytes
HES1: Hairy and Enhancer of Split 1
HEY1: Hairy/enhancer-of-split related with YRPW Motif Protein 1
HIF-1α: Hypoxia-inducible Factor-1α
HMGB1: High Mobility Group Box 1 Protein
HSP: Heat Shock Protein
HUVEC: Human Umbilical Vein Endothelial Cell
JAG1: Jagged 1
JAG2: Jagged 2
JAK2: Janus kinase 2
JNK: c-Jun N-terminal Kinase
PI3K: Phosphoinositide 3-kinase
PKC: Protein Kinase C
PKD: Protein Kinase D
PTEN: Phosphatase and Tensin Homolog
MAPK: Mitogen-Activated Protein Kinases
MINAR1: Major Intrinsically Disordered Notch2-Associated Receptor 1
MCP: Monocyte Chemoattractant Protein
MMPs: Matrix Metalloproteinases
mTOR: Mammalian role of the Rapamycin
NF-κB: Nuclear Factor Kappa B
NICD: Notch Intracellular Domain
Notch1: Notch Proteins 1
Notch2: Notch Proteins 2
Notch3: Notch Proteins 3
Notch4: Notch Proteins 4
NRP: co-Receptors Neuropilins
PDGF: Platelet-derived Growth Factor
PLGF: Placental Growth Factor
PMN: Pre-metastatic Niches
RGD: Arginine-glycine-aspartic acid peptide
TGF: Transforming Growth Factor
TNBC: Triple-Negative Breast Cancer
TNF: Tumor Necrosis Factor
VEGF: Vascular Endothelial Growth Factors
VEGFR: Vascular Endothelial Growth Factor Receptor
YBX1: Y-box binding protein 1
